# Growth, Root Formation, and Nutrient Value of Triticale Plants Fertilized with Biosolids

**DOI:** 10.1100/2012/467052

**Published:** 2012-04-19

**Authors:** Wendy Mercedes Rauw, Michael Bela Teglas, Sudeep Chandra, Matthew Lewis Forister

**Affiliations:** ^1^Departamento de Mejora Genética Animal, Instituto Nacional de Investigación y Tecnología Agraria y Alimentaria, 28040 Madrid, Spain; ^2^Department of Agriculture, Nutrition and Veterinary Sciences, University of Nevada, Mail Stop 202, Reno, NV 89557, USA; ^3^Department of Natural Resources and Environmental Science, University of Nevada, Mail Stop 186, Reno, NV 89512, USA; ^4^Department of Biology, University of Nevada, Reno, Mail Stop 314, Reno, NV 89557, USA

## Abstract

Biosolids are utilized as nutrient rich fertilizer. Little material is available on benefits to forage crops resulting from fertilization with biosolids. This paper aimed to compare the effects of fertilization with biosolids versus commercial nitrogen fertilizer on growth, root formation, and nutrient value of triticale plants in a greenhouse experiment. Per treatment, five pots were seeded with five triticale seeds each. Treatments included a nonfertilized control, fertilization with 100, 200, 300, 400, and 500 ml biosolids per pot, and fertilization with a commercial nitrogen fertilizer at the recommended application rate and at double that rate. Biomass production, root length, root diameter, nitrogen, phosphorus, and potassium concentration were analyzed at harvest. Fertilization with biosolids increased triticale production (*P* < 0.001); production was similar for the 100 to 400 mL treatments. Root length, nitrogen, and phosphorus concentration increased, and potassium concentration decreased linearly with application rate. At the recommended rate, biomass production was similar between fertilization with biosolids and commercial fertilizer. However, plants fertilized with commercial fertilizer had considerably longer roots (*P* < 0.001), higher nitrogen concentration (*P* < 0.05), and lower potassium concentration (*P* < 0.01) than those fertilized with biosolids. Our results indicate that at the recommended application rate, biomass production was similar between fertilization with biosolids and with commercial nitrogen fertilizer, indicating the value of biosolids fertilization as a potential alternative.

## 1. Introduction

Biosolids are derived from the treatment of domestic sewage sludge at publically owned treatment works. The term biosolids generally refers to sewage sludge treated to meet the land-application standards outlined in the Code of Federal Regulations, Title 40 (Part 503) under section 405 (d) of the United States Clean Water Act [1, 2]. Because of the increasing costs of sewage sludge disposal (e.g., landfilling) and the increasing desire to reuse waste residuals wherever possible, land application of biosolids is increasingly chosen as a disposal practice [3]. In addition, organic compounds, plant nutrients, and trace elements in biosolids make it a valuable resource for land application [4]. In North America, over half of the biosolids produced (approximately 3 to 4 million Mg) are applied to land as nutrient rich fertilizer [5, 6]. Much of this is used in agriculture, including animal production systems. The use of biosolids in animal production systems is widespread in North America as well as in other countries, such as the United Kingdom, Australia, New Zealand, and Pakistan. Biosolids can be used to produce forage and feed crops or can be used on pastures and range for grazing animals [7, 8]. Biosolids can impact domestic animals through feeding on vegetation grown on biosolid-amended soil or by direct consumption of the soil attached to vegetation [9].

 Despite the potential advantages of biosolid application in agriculture, land application of biosolids may potentially pose a risk to public health from heavy metals or toxic organics that might enter the food chain and from pathogens that might be present in the biosolids [10]. There is a considerable body of literature available on risk assessment of biosolids application (e.g., [11, 12]). However, less material is available on benefits to forage crops resulting from fertilization with biosolids. In the present study, Triticale (X *Triticosecale* Wittmack) is chosen as the subject of study due to its popularity as a forage crop in livestock production systems. It is a product of the cross between wheat (*Triticum, spp*.) and rye (*Secale cereale* L.), resulting in a crop that is environmentally more flexible than most other cereal crops and has been shown to have superior yields and tolerance to many diseases and pests relative to its parental species or distant relatives [13]. It can fulfill the needs of grazing, ensilage, hay, and grain for feed [14]. Breeding programs of triticale mainly focus on the improvement of economic traits such as yield, biomass, nutritional factors, plant height, early maturity, and grain volume weight [15]. Nutritional values of 15 high-yielding cultivars and lines of triticale were evaluated by Heger and Eggum [16]. The biological value of triticale protein was superior to that of wheat protein (65.3 versus 61.6); the utilizable protein yields for most triticale cultivars were higher than those for wheat [16]. The agronomic advantages of triticale grains over wheat make it an attractive option for increasing global food production, in particular for marginal and stress-prone growing conditions [15].

The aim of the present paper was to compare the influence of fertilization with biosolids versus fertilization with a commercial nitrogen fertilizer on growth, root formation, and nutrient value of triticale plants in a controlled greenhouse experiment.

## 2. Materials and Methods

### 2.1. Experimental Procedures

Class B biosolids from the Truckee Meadow Water Reclamation Facility, Reno, NV, were used in a greenhouse experiment between July and September 2009. Pots of 3.8 liters were seeded with 5 triticale (*Triticosecale)* seeds each. Data were collected on 5 pots for each of the following treatments: 0 (control), 100, 200, 300, 400, and 500 mL biosolids per pot, mixed with untreated soil originating from the Main Station Field Laboratory fields (MSFL; University of Nevada, Reno), and two treatments of Main Station Field Laboratory soil mixed with Best Ammonium Sulfate 21-0-0 commercial fertilizer at a recommended application rate of 10 mL (F1) per pot and at double that rate, 20 mL/pot (F2). The soil at the MSFL fields primarily consists of Truckee silt loam (fluvaquentic haploxeroll; [17]). Fertilizer application rates were calculated based on the estimated amount of plant-available nitrogen. According to the analytical report of the Truckee Meadow Water Reclamation Facility, biosolids contained 61792 mg/kg total Kjeldahl N, 10665 mg/kg Ammonium N, 313 mg/kg Nitrate N, and 51127 mg/kg Organic N. The amount of fertilizer needed to produce a yield goal of 7 tons/0.405 ha on MSFL soil was equivalent to 40.9 kilo liters of biosolids or 113 kg N fertilizer per 0.405 ha. This is translated to a recommended application rate of 185 mL of biosolids and 10 mL commercial fertilizer per pot. Therefore, the 200 mL biosolids treatment is comparable to recommended field application rates.

Plants were grown within a single greenhouse bay at the University of Nevada, Reno, under the same temperature and light intervals and all pots were watered daily until the plants were harvested for analysis. Plant height (HEIGHT) and number of leaves (LEAVES) were measured weekly for each individual plant until 4 weeks of age at which time they were harvested. Plant height was measured by stretching the tallest leaf on each plant to its full length. Growth rate (HRATE) and rate of leaf emergence (LRATE) were estimated by fitting a linear regression equation to data, averaged by pot, on HEIGHT and LEAVES as a function of age:

(1) $${\text{TRAIT}}_{\text{Age}}=a+\left(b\times \text{Age}\right),$$

where TRAIT_Age_ is the HEIGHT and LEAVES at a specific age (wk), *a* is the intercept, and *b* is the regression coefficient representing HRATE and LRATE per week, respectively.

 After harvest, plants were dried and above-ground plant biomass production was recorded for each pot (WEIGHT). Plant roots were separated from the soil, washed carefully, and individually analyzed using the WinRhizo 2007 root scanning program (Regent Instruments Inc, Montreal, Quebec), resulting in measurements of average root length (ROOTL) and diameter (ROOTD).

A single pot in the control group was excluded from the analysis due to abnormal plant growth. Of the remaining 195 plants, 9 plants were identified as exhibiting unusually slow growth (two plants from the 100 mL treatment, two from the 200 mL treatment, two from the 400 mL treatment, one from the 500 mL treatment, and two from the F2 treatment) both in terms of height and in terms of leaf emergence and were therefore not considered in the analyses of HEIGHT, LEAVES, HRATE, and LRATE. However, these plants could not be identified in the root data; therefore, root data was analyzed for the full dataset of 195 plants.

After harvest, plant samples were sent to A & L Western Agricultural Labs Inc (Modesto, CA, http://www.al-labs-west.com/) and analyzed for total nitrogen (by automated combustion at 900°C), and phosphorus and potassium concentration (by inductively coupled plasma emission spectrometry (ICP)) on dry matter (DM) basis. A & L Western Agricultural Labs Inc follows the North American Proficiency Testing (NAPT) Program (http://www.naptprogram.org/).

### 2.2. Data Handling and Statistical Analysis

The SAS program [18] was used for statistical analysis of all traits. The model used to describe the data on weekly, individual, measurements of HEIGHT, LEAVES, ROOTL, and ROOTD was as follows:

(2) $$Y_{ijk}=\mu +{\text{Treatment}}_{i}+{\text{Pot}\;(\text{Treatment})}_{j}+e_{ijk},$$

where *μ* is the population intercept, Treatment_*i*_ is the effect of treatment *i* (control, 100, 200, 300, 400, 500, F1, F2), Pot  (Treatment)_*j*_ is the effect of pot (1 to 5) nested within treatment *j*, and *e*_*ijk*_ is the residual error term of plant *k*, *e*_*ijk*_~NID(0,*σ*_*e*_^2^). The effect of treatment was considered fixed; the residual error term and the effect of pot nested within treatment were considered random. The traits HEIGHT, LEAVES, ROOTL, and ROOTD were denoted by *Y*_*ijk*_, as measured on plant k of treatment *i*, in pot *j*.

The model used to describe the data, averaged by pot, on HRATE, LRATE, and WEIGHT was

(3) $$Y_{ij}=\mu +{\text{Treatment}}_{i\;}+e_{ij},$$

where *μ*, Treatment_*i*_, and *e*_*ij*_ are as in model (1). The traits HRATE, LRATE, and WEIGHT were denoted by *Y*_*ij*_, as measured on plant *j* of treatment *i*.

Phenotypic correlations were calculated from measurements averaged by pot: HEIGHT and LEAVES at 4 weeks of age, HRATE, LRATE, WEIGHT, ROOTL, and ROOTD, and nitrogen, phosphorus, and potassium concentration, after adjusting the values for the effect of treatment with model (3).

## 3. Results

### 3.1. Plant Growth

**Figure 1:**
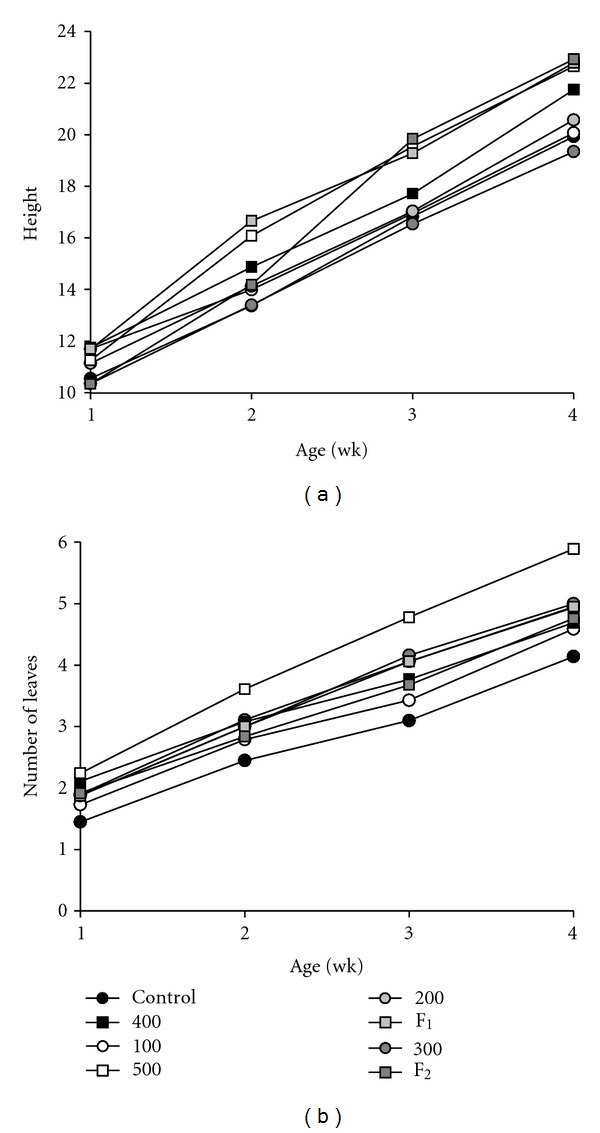
Average plant height (a) and number of leaves (b) from 1 to 4 weeks of age, by treatment (Control, 0, 100, 200, 300, 400, and 500 mL biosolids, and F1 and F2).

Figure 1 presents least squares means of plant height and leaf emergence between one and four weeks of age, adjusted for the effect of pot, for each treatment. The effect of treatment on HEIGHT and LEAVES was significant between two and four weeks of age (*P* < 0.001). At two weeks of age, plants from the 500 mL treatment were taller than those from the 0, 100, 200, 300 mL, and F2 treatments (*P* < 0.05), and plants from the F1 treatment were taller than those from the 0, 100, 200, 300, 400 mL, and F2 treatments (*P* < 0.05). At three weeks of age, plants from the 500 mL, F1 and F2 treatments were taller than those from the 0, 100, 200, 300, and 400 mL treatments (*P* < 0.05). At four weeks of age, plants from the 400 mL treatment were taller than those from the 300 mL treatment (*P* < 0.01) and plants from the 500 mL, F1, and F2 treatments were taller than those from the 0, 100, 200, and 300 mL treatments (*P* < 0.05).

At one week of age, plants from the control treatment had fewer leaves than those from the 400 and 500 mL treatments (*P* < 0.05) and plants from the 500 mL treatment had more leaves than those from the 100 mL treatment (*P* < 0.05). At two weeks of age, plants from the control line had fewer leaves than those from the 200, 400, and 500 mL treatments and plants from the 500 mL treatment had more leaves than those from the 100, 300 mL, F1, and F2 treatments (*P* < 0.05). At three weeks of age, plants from the control treatment had fewer leaves than those from the 200, 300, 500 mL, and F1 treatments (*P* < 0.01), plants from the 100 mL treatment had fewer leaves than those from the 300 and 500 mL treatments (*P* < 0.05), and plants from the 200, 400 mL, F1, and F2 treatments had fewer leaves than those from the 500 mL treatment (*P* < 0.05). At four weeks of age, plants from the control treatment had fewer leaves than those from the 200, 300, 500 mL, and F1 treatments (*P* < 0.05), and plants from the 500 mL treatment had more leaves than those from the 100, 200, 300, 400 mL, F1, and F2 treatments (*P* < 0.05).

Average HRATE, LRATE, WEIGHT, ROOTL, and ROOTD are presented in Table 1 for each treatment. Coefficients of determination (*R* ^2^) of curves fitting (1) per pot were 93 to 100% for HRATE and 79 to 100% for LRATE. Plants from the F2 group grew faster than those from the 100, 200, and 300 mL treatments. Biomass production was lower for the control treatment than that for all other treatments (*P* < 0.001), and higher for the 500 mL and F2 treatments than that for the 100 mL and F1 treatments (*P* < 0.05).

### 3.2. Root Development

Average root length increased with the level of biosolid application between 0 and 500 mL. A regression performed on root length as a function of the level of biosolid application gave a response of 12 cm per 100 mL biosolids with an *R* ^2^ of 81%.

The average root length in the control treatment was shorter than in all the other treatments (*P* < 0.05); average root length was shorter in the 100, 200, and 300 mL treatments than that in the 400, 500 mL, F1, and F2 treatments (*P* < 0.0001), shorter in the 400 mL treatment than that in the 500 mL, F1 and F2 treatments (*P* < 0.05), and shorter in the 500 mL treatment than that in the F1 and F2 treatments (*P* < 0.0001).

On average, roots were thinner in the control treatment than those in the 400, 500 mL, F1, and F2 treatments (*P* < 0.05), thinner in the 100 and 200 mL treatments than those in the 300, 400, 500 mL, F1, and F2 treatments (*P* < 0.05), thinner in the 300 mL treatment than those in the F1 treatment (*P* < 0.01), and thinner in the 500 mL treatment than those in the F1, treatment (*P* < 0.05).

### 3.3. Forage Nutrient Value

Average nitrogen and phosphorus concentration increased and average potassium concentration decreased with the level of biosolid application between 100 and 500 mL. A regression performed on nitrogen, phosphorus, and potassium concentration as a function of the level of biosolid application gave a response of 0.15, 0.06, and −0.35% change per 100 mL biosolids with an *R* ^2^ of 96, 95, and 89%, respectively.

Concentrations of nitrogen were lower in the control treatment than those in the 400, 500 mL, F1, and F2 treatments (*P* < 0.05), lower in the 100 mL treatment than those in the 300, 400, 500 mL, F1, and F2 treatments (*P* < 0.05), lower in the 200 and 300 mL treatments than those in the 500 mL, F1, and F2 treatments (*P* < 0.05), lower in the 400 mL treatment than those in the F1 and F2 treatments (*P* < 0.05), lower in the 500 mL treatment than those in the F2 treatment (*P* < 0.0001), and lower in the F1 treatment than those in the F2 treatment (*P* < 0.001).

Concentrations of phosphorus in the control treatment were higher than those in the 100 mL treatment (*P* < 0.05) and lower than those in the 400, 500 mL, and F2 treatments (*P* < 0.01). Phosphorus concentrations were lower in the 100 mL treatment than those in the 200, 300, 400, and 500 mL treatments, lower in the 200 mL treatment than those in the 400, 500 mL, and F2 treatments (*P* < 0.01), lower in the 300 mL treatment than those in the 500 mL, F1, and F2 treatments (*P* < 0.05), and lower in the 400 and 500 mL treatments than those in the F1 and F2 treatments (*P* < 0.0001).

Concentrations of potassium were higher in the control treatment than those in the 400, 500 mL, F1, and F2 treatments (*P* < 0.05), higher in the 100 and 200 mL treatments than those in the 300, 400, 500 mL, F1, and F2 treatments (*P* < 0.01), and higher in the 300 mL treatment than those in the F2 treatment (*P* < 0.05).

### 3.4. Phenotypic Correlations

Table 2 presents phenotypic correlations, adjusted for the effect of treatment, between HEIGHT and LEAVES at 4 weeks of age, HRATE, LRATE, WEIGHT, ROOTL, ROOTD, and nitrogen, phosphorus, and potassium concentration. Plants that grew taller grew at a faster rate, with a larger number of leaves that emerged at a faster speed, and produced more above-ground biomass. Plants with those properties had a lower nitrogen and phosphorus concentration. Plants that grew taller and at a faster rate had longer roots. Plants that had a lower potassium concentration were those that had a slower leaf emergence and tended to be those that had fewer leaves. Plants that had longer roots also had wider root diameters and a lower nitrogen concentration. Plants containing more nitrogen contained more phosphorus and plants containing more phosphorus contained more potassium.

## 4. Discussion

### 4.1. Biosolid Application Rate

In the present experiment, plants receiving the 500 mL biosolids treatment grew tallest with significantly more leaves, at the highest rate of leaf emergence. These traits resulted in a biomass production that was highest for the 500 mL treatment and indicates that in the present greenhouse experiment, fertilization with biosolids resulted in increased triticale production.

In contrast with growth traits, root length increased linearly with biosolid application rate, each 100 ml of biosolids adding 12 cm to their length. Root diameter increased with application rate, but this effect was not as pronounced. Plants with an increased root length grew faster and ended up being taller at four weeks of age. Tschaplinski and Blake [19] observed a positive relationship between early root production (number, length, and dry weight) and accumulation of aboveground biomass in hybrid poplar. In the present study, however, although taller plants produced more biomass, plants with longer roots had a higher biomass production but this was not significant. The relationship between application rate and root length did not translate to a linear effect in plant growth rate and length.

Plant nutrient concentration is determined by the stage of development, the species, variety or hybrid, the plant organ, and various parts or tissues of the organs. Among abiotic factors, application of fertilizers has the greatest effect on the nutrient concentration, in addition to genetic factors such as the capacity and dynamics of nutrient uptake and the utilization and distribution of assimilates [20]. Phosphorus concentration in the present experiment was higher than that observed by Brown et al. [21], who observed a triticale total P concentration of 0.18 to 0.53% with a mean of 0.33%. In the present experiment, increased levels of biosolid fertilization added 0.15% to the nitrogen concentrations and 0.06% to the phosphorus concentrations and subtracted 0.35% to the potassium concentrations for each 100 mL of biosolids added. Bennett et al. [22] observed that the application of nitrogen significantly increased the percent of nitrogen in corn leaves in all of eight experiments and that phosphorus percentage in the leaf was significantly increased in certain experiments. Also in the study of Lasztity [20], the P concentration increased simultaneously with a rising fertilizer dose (N, P, and K); the K concentration increased as the result of K application.

**Figure 2:**
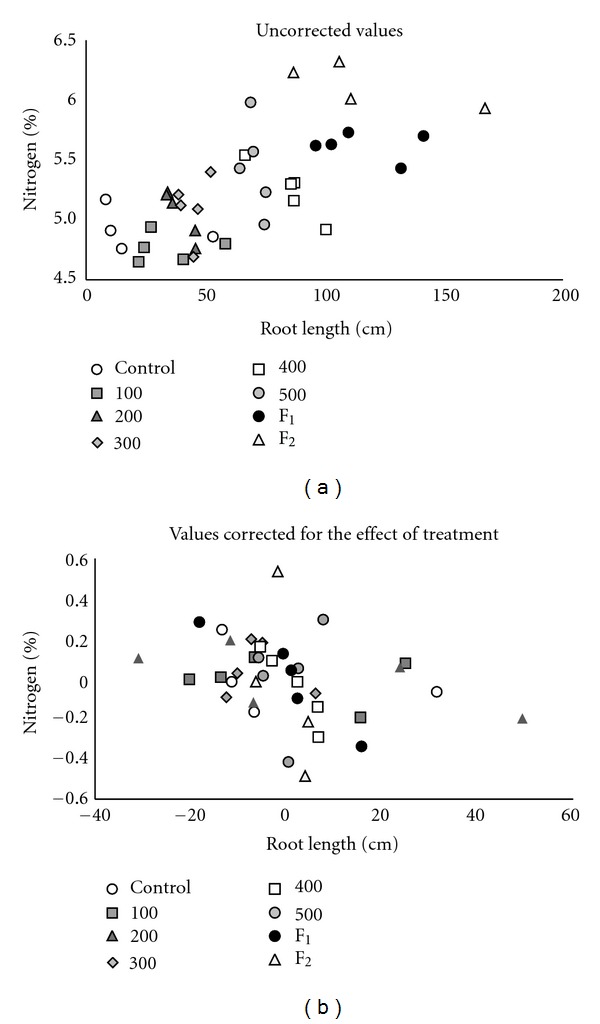
Relationship between root length and nitrogen concentration, by treatment (Control, 0, 100, 200, 300, 400, and 500 mL biosolids, and F1 and F2) (a), and relationship between root length and nitrogen concentration after correction for the effect of treatment, by treatment (Control, 0, 100, 200, 300, 400, and 500 mL biosolids, and F1 and F2) (b).

The configuration and growth rate of the root system influence nutrient uptake by plants [23]. This could explain the observation that both root length and the N and P concentration increased linearly with increasing biosolid application rates. However, root length was not significantly correlated with phosphorus or potassium concentration and was *negatively* correlated with nitrogen concentration, after adjustment for the effect of treatment. The relationship between root length and nitrogen concentration is depicted in Figure 2(a): although nitrogen concentration increases with increasing biosolid application rates and accompanying root lengths, it decreases with increasing root lengths within treatment, resulting in an overall negative correlation after adjustment for the effect of treatment (Figure 2(b)). In addition, a negative correlation was observed between growth traits and nitrogen, phosphorus, and potassium concentrations. This observation might be explained by the fact that concentrations of nutrients in triticale, such as N, P, K, Ca, and Mg, decrease during the vegetation period [24]. Therefore, although a higher rate of fertilization resulted in longer roots and an increased plant nutrient concentration, within application rate, faster growing plants with longer roots may be physiologically more mature resulting in a lower nutrient concentration when the results are adjusted for the effect of treatment.

### 4.2. Biosolids versus Commercial Fertilizer

In order to compare the effects of biosolid application with the application of commercial fertilizer, we compare the two recommended application rates, that is, the 200 mL biosolids treatment and the F1 commercial fertilizer treatment, with the control treatment. Our results indicate that growth rate and rate of leaf emergence were very similar for all three treatment groups, but that commercially fertilized plants were taller at four weeks of age than plants fertilized with biosolids and nonfertilized plants; nonfertilized plants had fewer leaves at four weeks of age than fertilized plants. Overall biomass production was significantly increased with fertilization but was similar for biosolids and commercial fertilizer treatments. This supports the findings by Priestly [25] who compared fertilization of wheat on agricultural land including two rates of biosolids and application of commercial fertilizers, Agstar and Urea. Results indicated no significant differences in grain yields between the treatments and it was concluded that biosolid application in wheat is likely to result in the same production levels as commercial fertilizers.

 However, in the present study, commercial fertilizer appeared to have a large positive effect on root length and root diameter. Those traits were considerably larger than in the control and 200 mL biosolid treatments. Nitrogen concentration was significantly higher and potassium concentration significantly lower in the F1 treatment compared with the control and 200 mL biosolid treatments. The two fertilizers had similar effects on the phosphorus concentration of the forage which was lower in both treatments than in the control treatment.

### 4.3. Double Dose Applications

In order to compare the influence of double-dose applications, we compare the two recommended applications rates of 200 mL biosolids and F1 commercial fertilizer with double the amount at 400 mL biosolids and F2 commercial fertilizer. Doubling the application rate did not significantly affect growth rate, rate of leaf emergence, or plant height and number of leaves at four weeks of age. However, overall biomass production was significantly higher in the F2 treatment than in the F1 treatment. Root length and root diameter were similar for the single application rates as for the double-application rates for both the biosolids treatment and the commercial fertilizer treatment. Doubling the application rate did not significantly influence nitrogen and potassium concentration of the triticale in the biosolids treatment or phosphorus and potassium concentration in the commercial fertilizer treatment, but it did result in higher phosphorus concentration in the biosolids treatment and higher nitrogen concentration in the commercial fertilizer treatment.

## 5. Conclusions

In conclusion, the results of the present greenhouse study indicate that fertilization with biosolids resulted in increased triticale production compared with nonfertilized plants; biomass production was very similar for the different application rates. Root length, nitrogen concentration, and phosphorus concentration increased, and potassium concentrations decreased linearly with application rate. At the recommended application rate, biomass production was similar between fertilization with biosolids and with commercial nitrogen fertilizer, indicating the value of biosolids fertilization as a potential alternative. However, plants fertilized with commercial fertilizer had considerably longer roots, higher nitrogen concentration, and lower potassium concentration than those fertilized with biosolids. In addition, preliminary results on a field study on triticale plants fertilized at the recommended application rate versus plants grown on non-fertilized fields indicate that fertilization with biosolids resulted in a lower dry mass and ash concentration, but higher N, crude protein, NDF, and ADF concentrations (results not presented). Further research is needed to verify all results in the field.

## Figures and Tables

**Table 1:** Least squares means and standard errors of the least squares means of growth rate (HRATE), rate of leaf emergence (LRATE), above-ground biomass production (WEIGHT), average root length (ROOTL), root diameter (ROOTD), and nitrogen, phosphorus, and potassium percentage on DM basis of *Triticosecale* plants, by treatment^1^.

	Control	100 mL	200 mL	300 mL	400 mL	500 mL	F1	F2	s.e.^2^	s.e.^3^
HRATE (cm/wk)	3.16^ab^	2.81^a^	3.12^a^	3.01^a^	3.28^ab^	3.77^ab^	3.59^ab^	4.34^b^	0.46	0.41
LRATE (number/wk)	0.87^a^	0.92^a^	1.01^a^	1.05^a^	0.85^a^	1.21^a^	1.03^a^	0.94^a^	0.15	0.14
WEIGHT (gr)	1.30^a^	4.07^b^	5.08^bc^	4.89^bc^	5.24^bc^	6.15^c^	4.46^b^	6.11^c^	0.59	0.53
ROOTL (cm)	21.8^a^	34.6^ab^	39.3^b^	44.6^b^	85.3^c^	70.8^d^	116.6^e^	117.9^e^	5.16	4.62
ROOTD (mm)	0.26^ab^	0.25^a^	0.23^a^	0.29^bd^	0.34^ce^	0.30^de^	0.33^ce^	0.31^e^	0.013	0.012
Nitrogen (%)	4.93^ab^	4.77^a^	5.05^abc^	5.10^bc^	5.25^cd^	5.43^de^	5.62^e^	6.14^f^	0.11	0.10
Phosphorus (%)	0.66^ad^	0.56^b^	0.68^ad^	0.74^ac^	0.78^ce^	0.82^e^	0.60^db^	0.54^b^	0.03	0.03
Potassium (%)	5.23^ab^	5.70^a^	5.68^a^	4.93^bc^	4.53^cd^	4.51^cd^	4.68^cd^	4.37^d^	0.20	0.18

^1^0 (control), 100, 200, 300, 400, and 500 mL biosolids mixed with untreated farm soil, and 5 mL (F1) and 10 mL (F2) commercial fertilizer mixed with untreated soil. ^2^Standard errors of the Control group. ^3^Standard errors of the 100, 200, 300, 400, and 500 mL biosolids groups and the F1 and F2 groups.

^a,b,c,d,e,f ^Values with different superscripts are significantly different (*P* < 0.05).

**Table 2:** Phenotypic correlations between plant height (HEIGHT) and number of leaves (LEAVES) at four weeks of age, growth rate (HRATE), rate of leaf emergence (LRATE), above-ground biomass production (WEIGHT), root length (ROOTL), root diameter (ROOTD), and nitrogen, phosphorus, and potassium percentage on DM basis in triticale plants.

	LEAVES	HRATE	LRATE	WEIGHT	ROOTL	ROOTD	Nitrogen	Phosphorus	Potassium
HEIGHT	0.59***	0.65***	0.47***	0.48***	0.17*	−0.09	−0.58***	−0.30***	−0.11
LEAVES		0.37***	0.64***	0.34***	0.06	0.09	−0.35***	−0.35***	−0.12^†^
HRATE			0.32***	0.35***	0.20**	−0.07	−0.51***	−0.21**	−0.06
LRATE				0.36***	0.07	−0.03	−0.50***	−0.44***	−0.16*
WEIGHT					0.09	−0.06	−0.33***	−0.25***	−0.03
ROOTL						0.26***	−0.19**	−0.06	−0.01
ROOTD							−0.05	−0.08	−0.02
Nitrogen								0.42***	0.11
Phosphorus									0.63***

*P < 0.05; **P < 0.01; ***P < 0.001.
